# Resveratrol Suppresses Cross-Talk between Colorectal Cancer Cells and Stromal Cells in Multicellular Tumor Microenvironment: A Bridge between In Vitro and In Vivo Tumor Microenvironment Study

**DOI:** 10.3390/molecules25184292

**Published:** 2020-09-18

**Authors:** Constanze Buhrmann, Parviz Shayan, Aranka Brockmueller, Mehdi Shakibaei

**Affiliations:** 1 Musculoskeletal Research Group and Tumor Biology, Chair of Vegetative Anatomy, Institute of Anatomy, Faculty of Medicine, Ludwig-Maximilian-University Munich, Pettenkoferstrasse 11, D-80336 Munich, Germany; constanze.buhrmann@med.uni-muenchen.de (C.B.); aranka.brockmueller@campus.lmu.de (A.B.); 2 Department of Parasitology, Faculty of Veterinary Medicine, University of Tehran, Tehran 141556453, Iran; pshayan@ut.ac.ir

**Keywords:** resveratrol, colorectal cancer, tumor microenvironment, paracrine cross-talk, CSC, NF-κB, TNF-β (lymphotoxin)

## Abstract

The interaction between tumor cells and the tumor microenvironment (TME) is an important process for the development of tumor malignancy. Modulation of paracrine cross-talk could be a promising strategy for tumor control within the TME. The exact mechanisms of multi-targeted compound resveratrol are not yet fully understood. Whether resveratrol can modulate paracrine signal transduction-induced malignancy in the multicellular-TME of colorectal cancer cells (CRC) was investigated. An in vitro model with 3D-alginate HCT116 cells in multicellular-TME cultures (fibroblast cells, T-lymphocytes) was used to elucidate the role of TNF-β, Sirt1-ASO and/or resveratrol in the proliferation, invasion and cancer stem cells (CSC) of CRC cells. We found that multicellular-TME, similar to TNF-β-TME, promoted proliferation, colony formation, invasion of CRC cells and enabled activation of CSCs. However, after co-treatment with resveratrol, the malignancy of multicellular-TME reversed to HCT116. In addition, resveratrol reduced the secretion of T-lymphocyte/fibroblast (TNF-β, TGF-β3) proteins, antagonized the T-lymphocyte/fibroblast-promoting NF-κB activation, NF-κB nuclear translocation and thus the expression of NF-κB-promoting biomarkers, associated with proliferation, invasion and survival of CSCs in 3D-alginate cultures of HCT116 cells induced by TNF-β- or multicellular-TME, but not by Sirt1-ASO, indicating the central role of this enzyme in the anti-tumor function of resveratrol. Our results suggest that in vitro multicellular-TME promotes crosstalk between CRC and stromal cells to increase survival, migration of HCT116 and the resveratrol/Sirt1 axis suppresses this loop by modulating paracrine agent secretion and NF-κB signaling. Fibroblasts and T-lymphocytes are promising targets for resveratrol in the prevention of CRC metastasis.

## 1. Introduction

The treatment of cancer still leads to varying, mostly unsatisfactory results, as the number of patients who develop chemoresistance to current therapies, relapses and metastasis is still a major problem [1]. Among most malignant cancers, colorectal cancer (CRC) remains a clinical challenge, ranking third in mortality and relapses [1]. CRC is a multi-step disease that is the sum of the accumulation of epigenetic and genetic changes stimulated by low-grade chronic inflammation [2,3].

Effective treatment of CRC poses such a major clinical challenge because the interaction between the tumor and its immediate environment, known as the tumor microenvironment (TME), is essential in the malignant progression of the tumor [4]. This cross-talk between cancer cells and stroma cells in TME is a key mechanism that stimulates and activates several signaling pathways, leading to tumor progression and malignancy, such as proliferation, migration, invasion and eventually metastasis [4,5], and for effective treatment the cross-talk in multicellular-TME between the cells must first be interrupted [6]. Key players in the multicellular-TME include tumor cells, recruited and transformed stromal fibroblasts, cancer-associated fibroblasts (CAFs) [7], and infiltrated immune cells such as B- and T-lymphocytes and macrophages, which have a broad spectrum of inflammatory functions and stimulatory effects on tumor cell progression [8]. It was described that cancer-associated stromal fibroblasts secrete cancer-stimulating factors such as growth factors and cytokines that transform the extracellular matrix and thus prepare the immediate TME of the tumor cells to facilitate metastasis [7]. It has been further described that immune cells in the gut play a fundamental role in maintaining tissue homeostasis and that dysregulation within immune cells stimulates chronic inflammation and may promote tumor development [9], and thus act as important prominent promoters of cancer progression [10]. In addition, stromal fibroblasts and immune cells in multicellular-TME actively contribute to tumor progression by cancer-induced cross-talk by evading the immunological response through the production of oxygen species and inflammatory cytokines [8,11,12].

It has been described that activation of the pro-inflammatory transcription factor NF-κB (nuclear factor kappa-light-chain-enhancer of activated B-cells) in tumor cells stimulates and promotes the cancer-mediated inflammatory response and paracrine signaling of pro-inflammatory cytokines and growth factors in TME, which in turn stimulates tumor progression and malignancy [13]. Under physiological conditions, NF-κB is closely involved in the biological process regulation of cell growth and survival as the main regulator of inflammatory and stress response [14]. In addition, constitutive NF-κB activation has been characterized in several cancers, which increases chronic low-grade inflammation and promotes cancer progression [15]. NF-κB promotes the production of pro-inflammatory cytokines, such as members of the family of tumor necrosis factors, TNF-α and TNF-β, in tumor cells, which further promote cancer cell progression [16,17,18,19,20]. It has been shown that NF-κB and NF-κB-dependent gene products in lymphocytes are up-regulated in TME [21], and the suppression of pro-inflammatory cytokine-mediated signaling pathways by inhibiting cross-talk between tumor cells and inflammatory cells could be a promising therapeutic strategy.

It is suggested that a subpopulation of cells in the tumor, cancer stem cells (CSCs), which have self-renewal capabilities, play a central role in mediating the cross-talk between tumor cells and TME [22]. These CSCs are located in the colon crypts, eventually replacing the normal stem cells and displaying a specific set of markers, including CD133+, CD 44+, CD166+ and ALDH+ [23]. There is evidence that the formation of CSCs is related to epigenetic modifications within the tumor cells and that the interaction between epigenetic mechanisms and the TME shapes the tumor architecture and regulates the plasticity of CSCs [24]. It is considered that chemoresistance and cancer recurrences are due to chemoresistant CSCs, and therapies that target the cross-talk of CSCs in cancer TME represent promising potential therapies [22,25].

Growth factors, such as the transforming growth factor-β (TGF-β), may initially be tumor suppressing, but later act as tumor promoters that facilitate tumor progression by stimulating proliferation, invasion and evasion of the immune system, and thus play a crucial role in cancer progression, since they are exchanged in TME and have paracrine and autocrine effects [26]. Here, TGF-β interacts with various signaling pathways, such as Wnt, Notch, mitogen-activated protein (MAP), kinase, phosphoinositide-3-kinase (PI3K) act, nuclear factor κB (NF-κB) and Janus kinase/signal transducers and activators of the transcription pathway (JAK/STAT) [27]. Interestingly, it has been described that TGF-β can act synergistically with pro-inflammatory cytokines such as TNF-α to activate the NF-κB signaling pathway [28].

Natural components are multi-targeting agents and have been described to improve conventional chemotherapy by targeting inflammatory responses in cancers, suppressing NF-κB activation, stimulating apoptosis and targeting CSCs, thereby overcoming monotherapy-associated failures and drug resistance [29,30]. More recently, natural products have come to the fore for therapeutic approaches to the treatment and prevention of cancer in the context of TME [31]. Resveratrol (trans-3,5,4’-trihydroxystilbene) is a natural stilbene found in a wide variety of foods such as peanuts, berries and red grapes and is believed to have anti-inflammatory, anti-oxidant and anti-tumor effects [32,33]. The mechanism of action of resveratrol in many diseases, both prophylactically and therapeutically, has not yet been clarified. Many in vitro experiments have shown positive results in many diseases and have been used to identify several direct subcellular molecular signaling targets for this compound, including Sirt1 [34,35]. Indeed, an important subcellular target for resveratrol-mediated suppression of colorectal cancer is the NAD+-dependent histone deacetylase sirtuin-1 (SIRT1) [36,37]. Sirt1 belongs to the class III histone/protein deacetylases (HDACs) known to regulate cellular metabolism, differentiation, inflammation, aging, apoptosis, proliferation and the cell cycle [38,39]. Moreover, in cancer, resveratrol has been shown to upregulate intercellular connections and focal adhesion molecules, block the epithelial-mesenchymal transition (EMT), suppress inflammatory pathways and increase apoptosis [37,40,41]. Recently, we showed that resveratrol chemosensitizes CRC cells to pro-inflammatory TNF-β-induced survival and proliferation by suppression of the NF-κB signaling axis [42]. In addition, there is increasing evidence that resveratrol can modulate the inflammatory and stress response in TME and thereby suppress cancer progression [43]. However, it has been reported that pharmacokinetic resveratrol is characterized as a rapidly absorbed compound that undergoes extensive metabolism by cytochrome P-450 and intestinal microbiota [44,45,46]. In addition, its metabolism in the body leads mainly to conjugation products whose biological activity is not yet known [47].

In this study, we show that resveratrol down-regulates CRC cell survival, colony formation, invasion and activation of CSC cells by targeting T-lymphocytes/fibroblasts and NF-κB signaling induced by TNF-β- or multicellular-TME, but not by Sirt1-ASO, pointing to the central role of this enzyme in the anti-tumor function of resveratrol in the suppression of cross-talk in pro-inflammatory multicellular-TME.

## 2. Results

**Figure 1:**
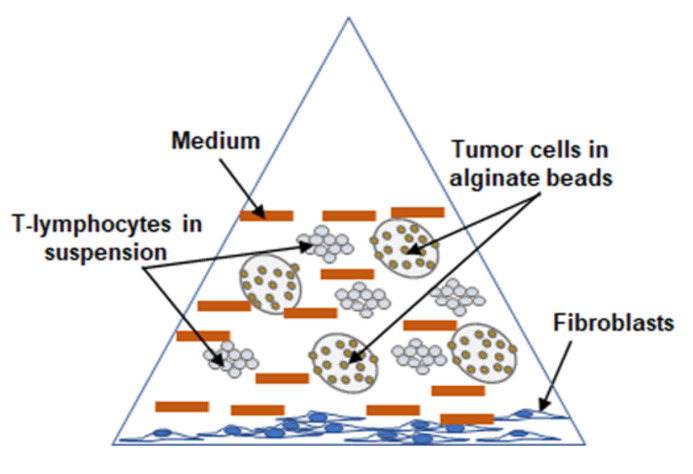
Schematic representation of the working model showing the experimental setup under pro-inflammatory multicellular-TME culture conditions.

The main focus of this study was to investigate and understand the functional significance of cross-talk in multicellular-TME of CRC (Figure 1) and to evaluate the ability of resveratrol in modulating targeted cross-talk between CRC cells and stromal cells in the TME.

### 2.1. Resveratrol Inhibits HCT116 Cell Proliferation Promoted by TNF-β- or Multicellular-TME, but Not by Sirt1-ASO

**Figure 2:**
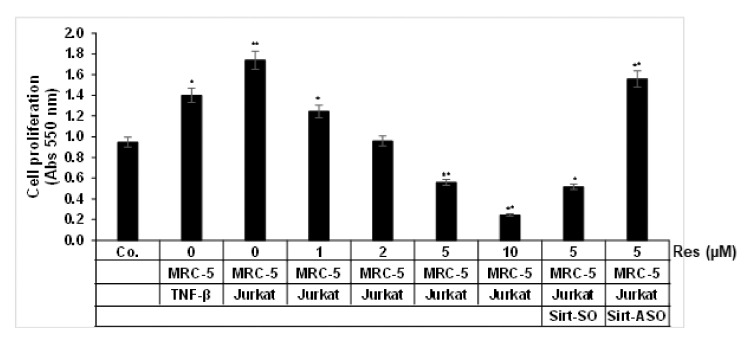
Impact of resveratrol and Sirt1-ASO on colorectal cancer cells (CRC) cell proliferation in the pro-inflammatory multicellular-TME. Serum-starved cultures of HCT116 cells in 3D alginate cultures alone (basal control = co) or co-cultured with fibroblasts and TNF-β (10 ng/mL) (TNF-β-TME) or co-cultured with fibroblasts and T-lymphocytes (pro-inflammatory multicellular-TME) were either left untreated, or multicellular-TME cultures were treated with various doses of resveratrol (Res) (1, 2, 5, 10 µM), or transfected with 0.5 µM sense oligonucleotide (SO) control, or antisense oligonucleotides (ASO) against Sirt1 in the presence of Lipofectin transfection reagent (10 µL/mL) and co-treated with resveratrol (5 µM) for 14 days in alginate cultures, and cell viability was measured using the MTT method, as described in the Materials and Methods section. All assays were performed at least three times. * *p* < 0.05, ** *p* < 0.01 relative to basal control.

First, we investigated the efficacy of resveratrol and/or Sirt1-ASO 3D-alginate HCT116 cultures in TNF-β or in multicellular-TME, mimicking the in vivo TME, and to elucidated the involvement of paracrine therapeutic mediators in intercellular crosstalk and on tumor cell proliferation and cell viability. Cells were either left untreated or treated with different concentrations of resveratrol in serum-starved medium for 14 days as described in the Materials and Methods section and evaluated using the MTT assay. TNF-β-TME or multicellular-TME significantly promoted the proliferation of CRC cells compared to untreated basal control by 47% and by 80%, respectively (Figure 2), indicating that the pro-inflammatory cytokine TNF-β- or multicellular-TME strongly stimulated the proliferation of HCT116 cells. Indeed, multicellular-TME almost doubled the proliferation of HCT116 compared to the basal control HCT116. In contrast, in multicellular-TME, resveratrol (1, 2, 5 and 10 μM) blocked the cell viability and proliferation of HCT116 cells in a dose-dependent fashion by 27%, 38%, 66% and 86%, respectively, compared to untreated multicellular-TME (Figure 2). Treatment of 3D-HCT116 alginate cultures in multicellular-TME with Sirt1-SO and resveratrol (5 µM) in the presence of Lipofectin (10 µL/mL) suppressed the cell viability and proliferation of HCT116 cells, similar to resveratrol treatment by itself by 70% (Figure 2) compared to multicellular- TME. However, transfection of 3D-HCT116 alginate multicellular-TME cultures with Sirt-ASO and resveratrol (5 µM) in the presence of Lipofectin for 14 days revealed promotion of the proliferation CRC cells similar to untreated multicellular-TME (Figure 2). Taken together, these data suggest that TNF-β, similar to multicellular-TME, can stimulate tumor cell viability and proliferation, thereby increasing the malignancy of cancer cells. The inhibition of this pro-inflammatory signaling pathway in multicellular-TME by resveratrol is dependent on the Sirt1 protein.

### 2.2. Resveratrol Inhibits HCT116 Cells Colonosphere Formation and Invasion Promoted by TNF-β- or Multicellular-TME, but Not by Sirt1-ASO 

**Figure 3:**
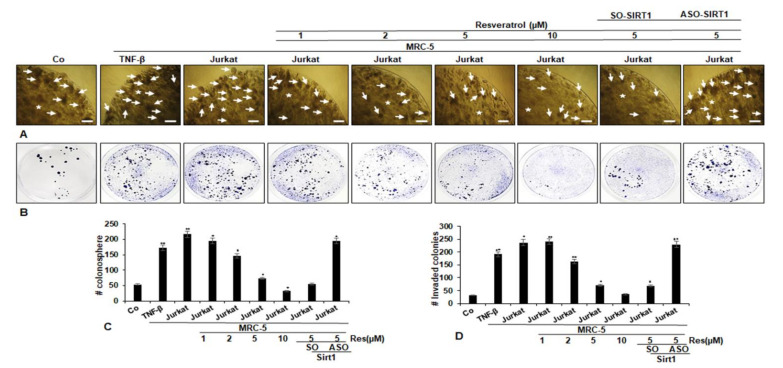
Impact of resveratrol and Sirt1-ASO on CRC cell colony formation and invasion in the multicellular-TME. Serum-starved cultures of HCT116 cells in 3D alginate cultures alone (basal control = co) or co-cultured with fibroblasts and TNF-β (10 ng/mL) (TNF-β-TME) or co-cultured with fibroblasts and T-lymphocytes (pro-inflammatory multicellular-TME) were either left untreated, or multicellular-TME cultures were treated with various doses of resveratrol (1, 2, 5, 10 µM), or transfected with 0.5 µM sense oligonucleotide (SO) control, or antisense oligonucleotides (ASO) against Sirt1 in the presence of Lipofectin transfection reagent (10 µL/mL) and co-treated with resveratrol (5 µM) for 14 days in alginate cultures (asterisks), as described in the Materials and Methods section. Colonosphere formation (**A**) and invasion (**B**) were evaluated by light microscopy. All experiments were performed at least three times. The number of colonospheres (arrows) and invaded colonies was quantified (**C**,**D**) by counting 20 different microscopic fields, and the number of attached colonies stained with toluidine blue, were quantified in each well. * *p* < 0.05, ** *p* < 0.01 relative to basal control. Magnification A: ×24, bar = 0.2 mm.

The impacts of resveratrol signaling on structural integrity, colonosphere formation and invasion, prominent features of cancer in TNF-β or in multicellular-TME and/or Sirt1-ASO were evaluated in 3D-alginate HCT116 cells. The 3D-alginate HCT116 cells in multicellular-TME were either left untreated or treated with different concentrations of resveratrol and/or Sirt1-ASO in serum-starved medium for 14 days, colonosphere formation and invasion evaluated using light microscopy as described in Materials and Methods. We found that resveratrol alone inhibited the colonosphere formation (Figure 3A) and invasion (Figure 3B) of HCT116 cells in a dose-dependent fashion. Furthermore, TNF-β- or multicellular-TME promoted the number of colonosphere formations and migrations clearly in HCT116 cells compared to that in basal control cultures (Figure 3A,B), suggesting the important role of this pro-inflammatory cytokine in TME in promoting the malignant potential of CRC cells. It was noted that there was no effect of Sirt1-SO on resveratrol-promoted inhibition of colonosphere formation and invasion on CRC cells. The measurement of colonosphere formation and migrated/adhered CRC colonies confirmed these findings. Moreover, knockdown of Sirt1 by mRNA abrogated the anti-tumor effects of resveratrol against CRC colonosphere formation, invasion and increased the number of colonosphere formations and migrated cells in the 3D alginate-culture in TME similar to control multicellular-TME (Figure 3C,D).

### 2.3. Resveratrol Suppresses Nuclear Translocation of p65-NF-κB in CRC Cells Stimulated by TNF-β- or Multicellular-TME, but Not by Sirt1-ASO

It has been reported that pro-inflammatory cytokines enhance the growth and infiltration of tumor cells by the transcription factor NF-κB [48]; for this reason, we examined the expression and nuclear translocation of NF-κB related to the malignancy and survival of CRC cells and conducted an immunofluorescence labelling for p65-NF-κB, as described in the Materials and Methods section.

**Figure 4:**
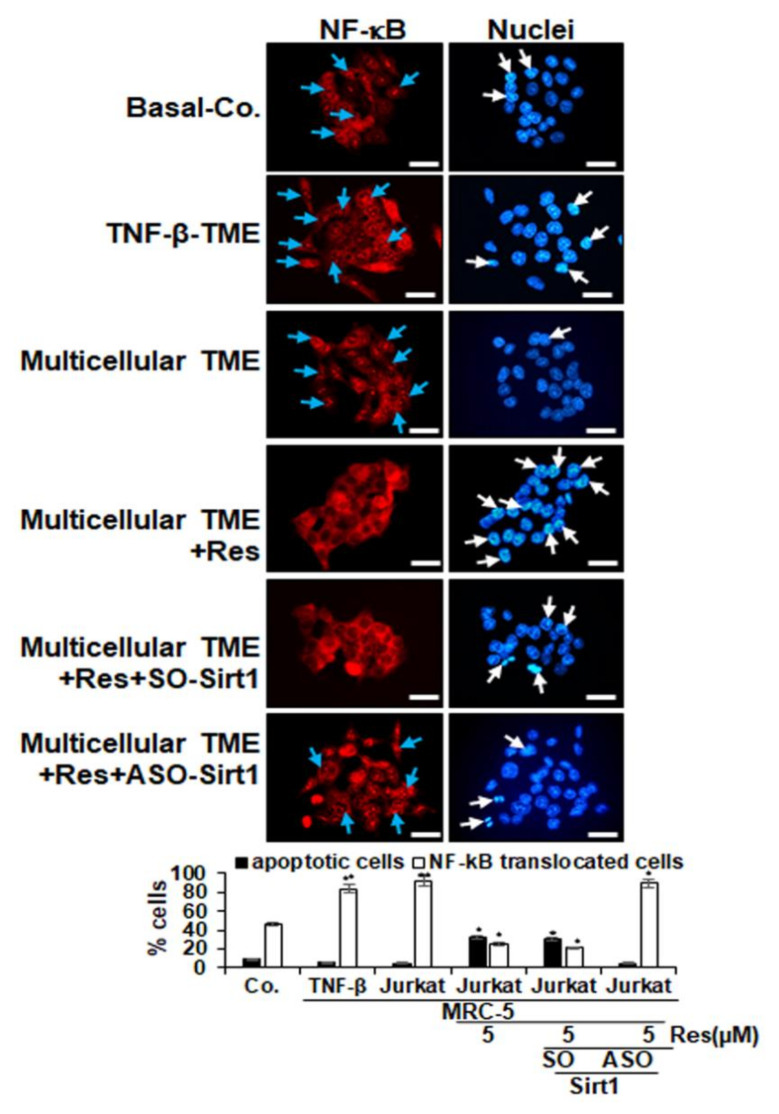
Impact of resveratrol and Sirt1-ASO on activation and nuclear translocation of p65-NF-κB in CRC cells in the multicellular-TME. Serum-starved HCT116 cells in monolayer cultures alone (basal control = co) or co-cultured with fibroblasts and TNF-β (10 ng/mL) (TNF-β-TME) or co-cultured with fibroblasts and T-lymphocytes (pro-inflammatory multicellular-TME) were either left untreated, or multicellular-TME cultures were treated with resveratrol (5 µM) for 4h in monolayer cultures, or transfected with 0.5 µM sense oligonucleotide (SO) control, or antisense oligonucleotides (ASO) against Sirt1 in the presence of Lipofectin transfection reagent (10 µL/mL) and co-treated with resveratrol (5 µM) for 4 h in monolayer cultures, as described in the Materials and Methods section. Magnification 600×; scale bar = 30 mm. All experiments were performed at least in triplicate and quantification of positively labelled p65-NF-κB-nuclei (blue arrows) and apoptotic nuclei (white arrows) were performed by counting 500–600 cells from 25 different microscopic fields (C). * *p* < 0.05, ** *p* < 0.01 relative to basal control.

In the untreated pro-inflammatory multicellular-TME control cultures, 92% of the HCT116 cells showed a strong label for p65-NF-κB in the cell nucleus. A similar signal was observed in the TNF-β-TME cultures (84%), while HCT116 cells as basal control showed a 46% nuclear label (Figure 4). Treatment with resveratrol alone resulted in a significant down-regulation of p65-NF-κB expression in CRC cells and nuclear localization in pro-inflammatory multicellular-TME cultures resulting in 25% positive labelled cells, indicating the important synergistic effects of paracrine cross-talk between HCT116, fibroblast cells and T-lymphocytes/TNF-β in supporting tumor promotion. In addition, transfection with Sirt1-ASO but not with the control Sirt1-SO blocked the resveratrol-promoted inhibition of p65-NF-κB in CRC cells and nuclear localization in CRC cell lines, underlining that Sirt1 is one of the major target proteins of resveratrol in resveratrol anti-tumorigenic effects in CRC cells (Figure 4). Taken together, these results show that resveratrol alone was capable of downregulating the expression of p65-NF-κB in multicellular-TME, one of the major mechanisms for inhibiting tumor growth and invasion.

In addition, we examined the amount of cell death by apoptosis by means of DAPI (4′,6-diamidino-2-phenylindole) labelling and fluorescence microscopy to understand the morphological changes of the cell nucleus (Figure 4). In the untreated proinflammatory multicellular- and TNF-β- TME cultures, HCT116 cells showed normal nucleus size and minimal morphological changes, resulting in 5% and 6% apoptosis, similar to basal control cultures (9%). In contrast, HCT116 cells co-treated with resveratrol exhibited 33% apoptotic nuclei in proinflammatory multicellular-TME cultures, showed a significant gain in fragmented nuclei and apoptotic morphological changes compared to control (Figure 4). Additionally, transfection with ASO-Sirt1 blocked resveratrol-induced apoptosis (5% apoptotic nuclei) but not transfection with control SO-Sirt1 (30% apoptotic nuclei). These results are consistent with data from the MTT assay and show that resveratrol is able to block the pro-cancer effects of pro-inflammatory TME in CRC cells by suppressing proliferation and enhancing apoptosis (Figure 4).

### 2.4. Resveratrol Modulates Suppression of Sirt1 Expression, NF-κB Phosphorylation and Biomarkers Linked with Inflammation, Invasion, Proliferation and Apoptosis in HCT116 Cells Induced by TNF-β- or Multicellular-TME, but Not by Sirt1-ASO

Considering that activation of the pro-inflammatory transcription factor NF-κB signaling is one of the major pathway activations of pro-inflammatory TME in CRC cells [15,49], the HCT116 cells were cultured in 3D-alginate beads and co-cultured in the multicellular-TME, TNF-β-TME and then either left untreated or treated with various doses of resveratrol (1, 2, 5, 10 µM), or were transfected with Sirt1-SO, Sirt1-ASO (0.5 µM) in the presence of Lipofectin (10 µL/mL) and co-treated with resveratrol (5 µM) for 14 days in serum-starved medium, as described in the section Materials and Methods. Whole cell lysates were subjected to immunolabeling with antibodies against Sirt1, p65, phospho-specific p65-NF-κB and NF-κB-regulated gene products involved in invasion (MMP-9), metastasis (CXCR4) and proliferation (Ki-67) and apoptosis (cleaved-caspase-3). In addition, HCT116 cells alone in alginate microenvironment were used as basal control.

**Figure 5:**
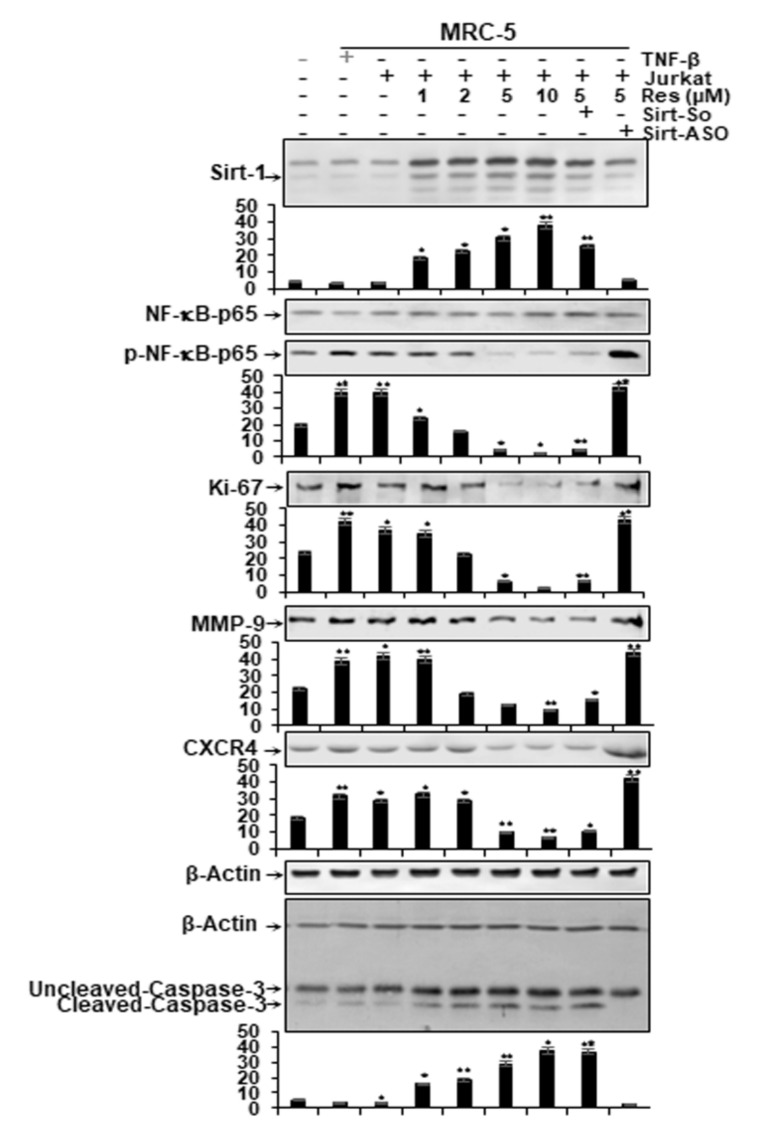
Impact of resveratrol and Sirt1-ASO on Sirt1 expression, stimulation of NF-κB and NF-κB-promoted gene end-products in CRC cells in the multicellular-TME. Serum-starved cultures of HCT116 cells in 3D alginate cultures alone (basal control = co) or co-cultured with fibroblasts and TNF-β (10ng/mL) (TNF-β-TME) or co-cultured with fibroblasts and T-lymphocytes (pro-inflammatory multicellular-TME) were either left untreated, or multicellular-TME cultures were treated with various doses of resveratrol (1, 2, 5, 10 µM), or transfected with 0.5 µM sense oligonucleotide (SO) control, or antisense oligonucleotides (ASO) against Sirt1 in the presence of Lipofectin transfection reagent (10 µL/mL) and co-treated with resveratrol (5 µM) for 14 days in alginate cultures, as described in the Materials and Methods section. Whole cell lysates were prepared and analyzed by Western blotting with antibodies against Sirt, p65-NF-κB, phospho-p65-NF-κB, MMP-9, CXCR4, Ki67 and cleaved-caspase-3. β-actin served as an internal loading control in all experiments. * *p* < 0.05, ** *p* < 0.01 relative to basal control.

In the multicellular-TME, similar to the TNF-β-TME, the expression of Sirt1 protein was down-regulated, similar to basal control (Figure 5). Treatment of the multicellular-TME with resveratrol alone and/or Sirt1-SO up-regulated the expression of Sirt1 protein in a dose-dependent manner (Figure 5) in CRC cells. In contrast, treatment with Sirt1-ASO significantly down-regulated levels of Sirt1 protein in CRC cells (Figure 5), underlining a crucial role for Sirt1 in resveratrol-inducing anti-tumorigenic effects in CRC cells.

Additionally, we found a significantly higher level of the p65-NF-κB and of the mentioned NF-κB-supported gene products expression of HCT116 alginate in the proinflammatory multicellular- TME and the TNF-β-TME versus the HCT116 alginate cultures of the basal control. Further, resveratrol significantly reduced pro-inflammatory multicellular-TME culture or TNF-β-TME induced phosphorylation of p65-NF-κB and the expression of the mentioned NF-κB-supported gene products in HCT116 cells in a concentration-dependent fashion (Figure 5). However, transfection with specific ASO against Sirt1 and co-treated with resveratrol abrogated effects of resveratrol and induced phosphorylation of NF-κB and the expression of the mentioned NF-κB-supported gene products. Co-treatment with resveratrol suppressed NF-κB activation and the expression of the mentioned NF-κB-supported biomarkers in Sirt1-SO-treated but not in Sirt1-ASO-treated cells, indicating that Sirt1 suppression to mRNA levels is not reversible by resveratrol, highlighting the essential role of Sirt1 in inhibiting the NF-κB signal pathway (Figure 5). We further investigated whether resveratrol can modulate NF-κB-dependent gene products induced by apoptosis (cleavage of caspase-3) in multicellular-TME and/or TNF-β-treated CRC cells. As shown in Figure 5, resveratrol clearly promoted caspase-3 cleavage in a concentration-dependent fashion in HCT116 cells in the pro-inflammatory multicellular-TME and the TNF-β-TME versus the HCT116 alginate cultures of the basal control (Figure 5). Transfection with specific Sirt1-ASO, but not with control Sirt1-SO, inhibited the resveratrol-induced cleavage of caspase-3 in CRC cells in multicellular pro-inflammatory TME cultures (Figure 5), indicating that Sirt1 suppression to mRNA levels is not reversible by resveratrol and Sirt1 is one of the major targeting proteins by resveratrol during resveratrol-induction-apoptosis effects in CRC cells. Taken together, these findings suggest that the down-regulation of Sirt1 with specific ASO downregulated Sirt1 protein levels and Sirt1-NF-κB complex formation during tumorigenesis in alginate cultures (Figure 5), thereby blocking the ability of resveratrol to phosphorylate NF-κB, which may at least partially inhibit resveratrol-promoting antitumorigenic effects in CRC cells. Taken together, these results suggest that knock-down of Sirt1 protein with specific ASO down-regulates the formation of Sirt1-NF-κB complexes during tumorigenesis in alginate cultures (Figure 5), and thereby blocks the ability of resveratrol to inhibit NF-κB, which may at least partially inhibit resveratrol-promoting anti-tumorigenic effects in CRC cells.

### 2.5. Resveratrol Suppresses Cancer Stem Cells in CRC Cell Populations Stimulated by TNF-β- or Multicellular-TME, but Not by Sirt1-ASO

**Figure 6:**
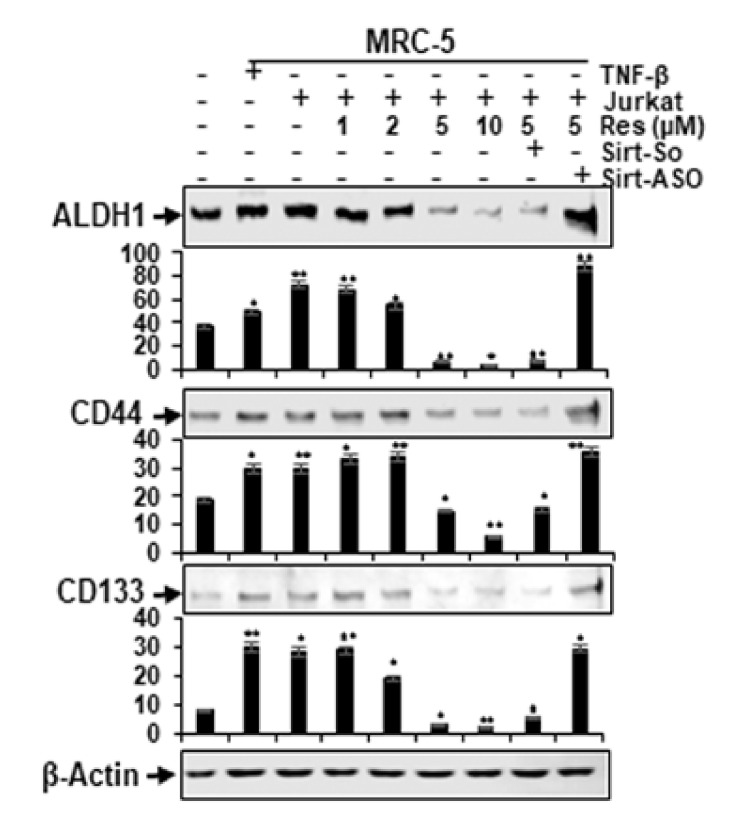
Impact of resveratrol and Sirt1-ASO on activation of cancer stem cells in the multicellular- TME. Serum-starved cultures of HCT116 cells in 3D alginate cultures alone (basal control = co) or co-cultured with fibroblasts and TNF-β (10 ng/mL) (TNF-β-TME) or co-cultured with fibroblasts and T-lymphocytes (pro-inflammatory multicellular-TME) were either left untreated, or multicellular-TME cultures were treated with various doses of resveratrol (1, 2, 5, 10 µM), or transfected with 0.5 µM sense oligonucleotide (SO) control, or antisense oligonucleotides (ASO) against Sirt1 in the presence of Lipofectin transfection reagent (10 µL/mL) and co-treated with resveratrol (5 µM) for 14 days in alginate cultures, as described in Material and Methods. Whole cell lysates were prepared and analyzed using Western blotting with antibodies against CD133, CD44 and ALDH1 in HCT116 cells. β-actin served as an internal loading control in all experiments. * *p* < 0.05, ** *p* < 0.01 relative to basal control.

TME has already been shown to play an important role in the induction and sustaining of cancer stem cells (CSCs) affected by stroma, inflammatory cells, cytokines and growth factors secreted by stroma fibroblasts [23]. For this reason, we examined the cellular susceptibility of CSCs within the CRC cell population, and pro-inflammatory multicellular-TME cultures of HCT116 cells were either left untreated or treated as described in the Materials and Methods in this report. In a further series of experiments, HCT116 cells were used alone in an alginate microenvironment as a basal control. We evaluated the levels of expression of CSC biomarkers (CD133, CD44 and ALDH1) on tumorigenicity and the effect of resveratrol in a concentration-dependent fashion on these CSC biomarkers. Control alginate microenvironment cultures (excluding MRC fibroblasts or T-lymphocytes) of HCT116 cells showed basal levels of expression of CSC biomarkers (Figure 6). In contrast, however, immunoblotting studies showed remarkably upregulated concentrations of CD133, CD44 and ALDH1 in HCT116 cells from multicellular-TME, similar to TNF-β-TME (Figure 6). Interestingly, treatment with resveratrol and co-treatment with Sirt1-SO-treated multicellular-TME cultures significantly down-regulated the expression of the mentioned CSC markers in a concentration-dependent manner. In contrast, the knockdown of Sirt1 with Sirt1-ASO resulted in the elimination of the blocking effect of resveratrol on the expression of mentioned CSC biomarkers, indicating the excellent targeting of resveratrol on CSCs (Figure 6) and the important role of Sirt1 in resveratrol-enhancing anti-tumorigenic effects in CRC cells in multicellular-TME. The densitometric evaluation of representative Western blot experiments revealed and approved the above described results (Figure 6). In summary, these results suggest that a further anti-tumor effect of resveratrol is mediated by down-regulation of the CSC signaling pathway and also by inhibition of NF-κB activation in CRC, even in multicellular-TME.

### 2.6. Resveratrol Inhibits Intensive Crosstalk between HCT116 Cells and Stromal Cells Induced by TNF-β- or Multicellular-TME, but Not by Knockdown of Sirt1 with Sirt1-ASO 

It has been reported that the secretion of pro-inflammatory cytokines and growth factors such as TGF-β3 by stromal cells into the tumor microenvironment leads to tumor growth and malignancy in various organs, which supports drug resistance, tumor recurrence, invasion and metastasis of neoplastic cells [50,51,52]. To further investigate the possible role of paracrine components in the process of synergistic crosstalk in the interaction of cancer and stroma cells in pro-inflammatory multicellular-TME, we next analyzed TGF-β3 and TNF-β expression in HCT116 to find out whether TGF-β is implicated in the boost of tumor cell proliferation and tumor promoting factors.

HCT116 cells in pro-inflammatory multicellular-TME or TNF-β-TME cultures were either left untreated or treated as described in the Materials and Methods section. In a further series of experiments, HCT116 cells were cultivated alone in an alginate microenvironment as a basal control. We investigated the expression levels of the proteins TGF-β3 and TNF-β on tumorigenicity and the effect of resveratrol in a concentration-dependent manner on these biomarkers.

**Figure 7:**
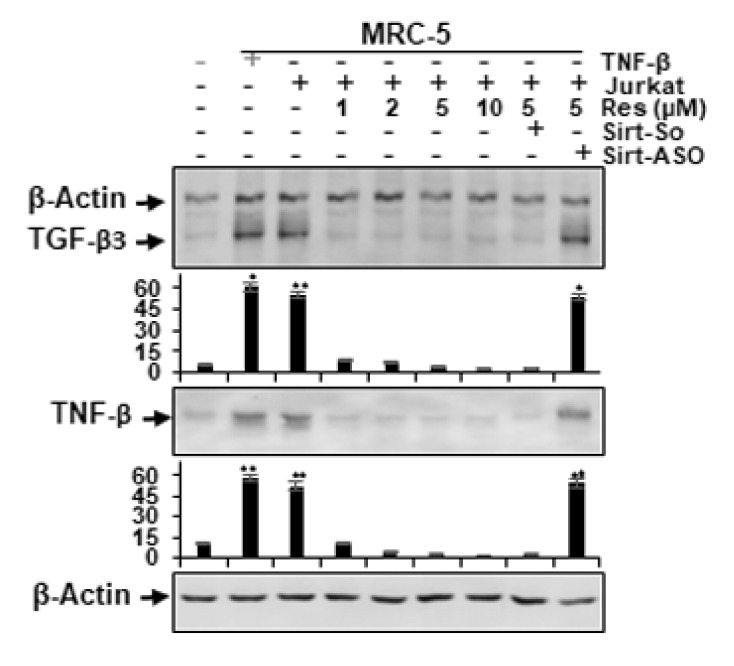
Impact of resveratrol and Sirt1-ASO on TNF-β and TGF-β3-mediated crosstalk between HCT116 cells and stromal cells in the multicellular-TME. Serum-starved cultures of HCT116 cells in 3D alginate cultures alone (basal control = co) or co-cultured with fibroblasts and TNF-β (10 ng/mL) (TNF-β-TME) or co-cultured with fibroblasts and T-lymphocytes (pro-inflammatory multicellular-TME) were either left untreated, or multicellular-TME cultures were treated with various doses of resveratrol (1, 2, 5, 10 µM), or transfected with 0.5 µM sense oligonucleotide (SO), or antisense oligonucleotides (ASO) against Sirt1 in the presence of Lipofectin transfection reagent (10 µL/mL) and co-treated with resveratrol (5 µM) for 14 days in alginate cultures, as described in Material and Methods. Whole cell lysates were prepared and analyzed by Western blotting with antibodies against TNF-β and TGF-β3. β-actin served as an internal loading control in all experiments. * *p* < 0.05, ** *p* < 0.01 relative to basal control.

Control alginate microenvironment cultures (without fibroblasts or T- lymphocytes) of HCT116 cells showed basal expression levels of TGF-β3 and TNF-β proteins (Figure 7). In contrast, immunoblotting experiments showed strikingly increased levels of TGF-β3 and TNF-β in HCT116 cells from multicellular-TME, similar to TNF-β-TME (Figure 7). Importantly, treatment with resveratrol and co-treatment with Sirt1-SO-treated multicellular-TME cultures significantly down-regulated the expression of the above-mentioned proteins in a concentration-dependent manner. In contrast, the knockdown of Sirt1 with Sirt1-ASO in multicellular-TME resulted in the elimination of the blocking effect of resveratrol on the expression of the mentioned biomarkers, indicating the superior targeting of resveratrol (Figure 7) and the leading role of Sirt1 in resveratrol-promoting anti-cancer activity in CRC cells even in multicellular-TME. These results are consistent with other studies showing that TGF-β and pro-inflammatory cytokines are the dominant paracrine triggers within the tumor microenvironment [53,54]. Taken together, these results suggest that paracrine crosstalk between tumor and stromal cells is crucial for the induction of tumor progression, invasion and metastasis and that the resveratrol/Sirt1 signaling pathway in pro-inflammatory TME co-cultures has a strong suppressive effect on this interaction.

## 3. Discussion

In the current research, we show that resveratrol can block cross-talk between HCT116 CRC cells, fibroblast MRC-5 cells and T-lymphocytes in a 3D alginate pro-inflammatory multicellular-TME model (Figure 1) that better mimics hybrid pro-inflammatory TME in vivo. Moreover, a large number of reports have previously shown that specific cross-talk in the tumor microenvironment between tumor cells and stromal cells can induce the growth, survival, proliferation, invasion and malignancy behavior of tumor cells and their ability to generate drug chemoresistance [55,56,57].

In this report, we investigated for the first time the specific cross-talk between 3D alginate HCT116 cells co-cultured with fibroblasts MRC-5 cells and T-lymphocytes in multicellular-TME. We found that (1) a dynamic interactive process took place between three cells, significantly increasing proliferation, colony formation and invasion of CRC cells. (2) TNF-β- or multicellular-TME-induced activation of NF-κB and NF-κB-regulated gene end products involved in proliferation (Ki-67), invasion (MMP-9), metastasis (CXCR4) and apoptosis (cleavage of caspase-3) in CRC cells. (3) Interestingly, we found that resveratrol suppressed the proliferation, colony formation and invasion of CRC cells in 3D alginate cultures in multicellular-TME, and this was effectively blocked by the specific knockdown of Sirt1 through ASO-Sirt1, pointing to the fact that Sirt1 suppression at the mRNA level is not reversible, emphasizing the crucial role of this enzyme. (4) Resveratrol suppressed the TNF-β- or multicellular-TME-induced formation of CSC-like cells. (5) The intense cross-talk in the co-cultures of multicellular-TME increased TGF-β3 and TNF-β levels in HCT116 cells compared to basal control, indicating an active TGF-β3 and TNF-β signal in tumor cells of multicellular-TME. (6) Finally, the expression of TGF-β3 and TNF-β in HCT116 cells was significantly reduced by treatment with resveratrol.

Our results are consistent with the results of other groups that have previously demonstrated that the specific cross-talk of tumor cells and stromal cells is a key factor in the initiation and progression of tumors by secreting and exchanging cytokines and growth factors in the specific tumor microenvironment, that imitate the interaction between these cells in the microenvironment of the tumor in vivo [58,59,60]. We found that transcription factor NF-κB is one of the most important activated down-stream signaling pathways for the TNF-β and the pro-inflammatory multicellular-TME and resveratrol/Sirt1 signaling has a specific modulatory effect against TNF-β-or multicellular-TME-induced phosphorylation of NF-κB and NF-κB-promoted gene end-products in CRC cells. These results are consistent with other studies that have shown that the pro-inflammatory transcription factor NF-κB is up-regulated in its response to various pro-inflammatory agents such as cytokines, mitogens, bacterial products, viral proteins and apoptosis-inducing compounds [61,62]. Under normal conditions, NF-κB is present in the cytoplasm as an inactive heterotrimer protein and upon activation, the degradation of IκBa releases nuclear localization signals on the NF-κB, resulting in nuclear translocation and binding to a specific sequence in the DNA, which in turn leads to transcription of the gene. Indeed, NF-κB is an ideal candidate for the development of anti-cancer drugs, since the triggering of NF-κB in tumor cells has been shown to block apoptosis and induce proliferation [63,64]. Pro-inflammatory TME can induce the mobilization of NF-κB [65], and the release of NF-κB leads to resistance to chemotherapeutic agents [66,67], and most importantly, molecules involved in tumor initiation, tumor promotion and metastasis are regulated by NF-κB [68]. For this reason, compounds that can block the triggering of the transcription factors NF-κB have the potential to prevent tumor initiation, promotion and metastasis.

It has been reported that resveratrol is known to be a multi-targeted safe, plant-derived polyphenolic compound [34] and has been associated with anti-inflammatory [69], anti-oxidant [70], anti-depressant [71], anti-atherogenic [72], anti-aging [73], as well as anti-cancer [74,75,76] properties, but the mechanisms of the complex action of resveratrol signaling during carcinogenesis in multicellular-TME are not yet fully understood. Furthermore, our group has published extensively on the anti-inflammatory and anti-apoptotic properties of resveratrol in various CRC tumor cells in monolayer and 3D alginate cultures, often showing how the resveratrol signaling pathway is dependent on Sirt1 as one of its important intracellular target proteins [17,18,36,37,41,42]. However, this work is the first to show how resveratrol exhibits an anti-proliferative and invasive effect in an in vivo similar pro-inflammatory multicellular-TME. To investigate the underlying mechanism of sensitivity of CRC cells to resveratrol in multicellular-TME, we investigated whether the effects of resveratrol on growth and metastasis of 3D-CRC alginate cultures in multicellular-TME were associated with inhibition of NF-κB activity. Several indications suggest that NF-κB mediates tumor progression, and it is known that tumor chemoresistance of various tumor cells induces NF-κB activation [49,50]. Moreover, our group has previously shown that co-treatment with resveratrol reduced NF-κB activation in IL-1β but not in Sirt1-ASO treated normal or cancer cells in monolayer cultures, indicating that Sirt1 suppression is not reversible at the mRNA level, emphasizing the important role of Sirt1 in the resveratrol specific signaling pathway [36,39]. Indeed, it was reported that down-regulation of Sirt1 at least partially suppressed the effects of resveratrol-promoting chemopreventive and anti-tumorigenic effects in CRC cells [36]. In addition, resveratrol has also been shown to downregulate inflammation in colorectal cancer by suppressing the expression of SUMO1 (small ubiquitin-like modifier protein 1) and nuclear translocation, thereby suppressing the Wnt/β catenin pathway [77].

The current study supports this idea that the induction of proliferation, colony formation and invasion of CRC cells in multicellular-TME strongly suggests that high expression of pro-inflammatory T-lymphocyte factors (cytokines, such as TNF-β) and high expression of growth factors from fibroblasts, such as TGF-β3, induce tumorigenesis and thus metastasis of CRC cells. In addition, resveratrol suppressed proliferation, colony formation and invasion in combination with its subcellular protein Sirt1, acting as a targeted inhibitor of multicellular-TME-induced malignancy of CRC cells. Interestingly, the suppressive effect of resveratrol is attenuated by the knockdown of the Sirt1 protein via ASO, which shows that the inhibition of Sirt1 is not reversible at the mRNA level, which underlines the specific resveratrol/Sirt1 signaling pathway in this process. These results are consistent with studies reporting that resveratrol can suppress the proliferation and growth of gastric cancer cells and CRC in a Sirt1-dependent manner in vitro and in vivo [36,78]. However, in this study we have shown for the first time the suppression of CRC cell proliferation, colony formation and invasion by the resveratrol/Sirt1 pathway in a 3D alginate culture in multicellular-TME.

Moreover, several lines of evidence have shown that the influence of T-lymphocyte phenotypes on the tumor depends mostly on the type of tumor together with its severity status and whether the cancer is a chronic inflammatory disease, which may explain the contradictory statements. In the case of CRC, which is associated with chronic inflammation, an anti-inflammatory response might be more beneficial, at least in the early stages of the disease, which may explain why resveratrol is seen to be an effective treatment for CRC cancer in vitro and in vivo [36,79], and thus, partly due to the ability of the resveratrol/Sirt1 axis to change from a pro-inflammatory T-lymphocyte response to an anti-inflammatory effect in the multicellular-TME.

We have further shown that multicellular-TME accelerates the progression and metastasis of CRC cells by activating cancer stem cells (CSC). Resveratrol suppresses the activation of CSC cells, which explains the high and specific targeting of resveratrol for CSC, as one of its anti-tumor effects. In addition, these results suggest that the other anti-tumorigenic effects of resveratrol are partly mediated by the down-regulation of the CSC signaling pathway and these data suggest further that the paracrine cross-talk between CRC tumor cells and stromal cells is important in inducing CSCs and treatment with resveratrol and Sirt1-SO down-regulated the expression of specific CSC biomarkers. In contrast, treatment with Sirt1-ASO clearly up-regulated levels of specific CSC biomarkers in a dose-dependent manner, highlighting the essential role for Sirt1 in resveratrol-promoting anti-tumorigenic effects in CRC cells. Indeed, these results are consistent with other studies that demonstrated that CSCs are responsible for proliferation, progression, metastasis and tumor recurrence in tumor cell population, but they make up only a small fraction of cancer cells and they are activated in pro-inflammatory TME [80,81].

We also found intensive cross-talk in multicellular-TME during tumor co-cultures and increased TGF-β3 and TNF-β expression in HCT116 cells, underlining active TGF- β3 and TNF-β signaling as paracrine interactions between T-lymphocytes, fibroblasts and HCT116 cells in the TME. In addition, the expression of paracrine agents such as TGF-β3 and TNF-β was significantly blocked by treatment with resveratrol in multicellular-TME. Interestingly, TGF-β3 has been reported to be able to stimulate NF-κB and related pro-inflammatory proteins in tumor cells [82]. Moreover, these results are consistent with other studies showing that TGF-β3 is the most prominent of the paracrine factors within the tumor microenvironment [53,54]. Furthermore, these results are in accordance with the previous findings from our laboratory that 1) TGF-β3 secretion as an important potential paracrine agent was found to play an integral role in microenvironment co-culture, 2) the expression of TGF-β3 and the phosphorylation of nuclear p-Smad2 was significantly inhibited by the neutralization of pan-TGF-β antibodies, pointing to a strong TGF-β dependency [83].

In addition, pro-inflammatory cytokines such as IL-1β, TNF-α, and TNF-β (Lymphotoxin-α) have been associated with chronic inflammation and tumorigenesis, and several reports from our and other laboratories have shown a strong correlation between chronic inflammation and cytokines, which controls the microenvironment of the tumor through modulation of the survival and migration of tumor cells as well as surrounding cells and thus induce tumor progression and metastasis [18,20,42,84,85,86]. However, the underlying role of molecular TNF-β and resveratrol signal transduction during tumorigenesis is still not fully understood. In fact, recent studies from our laboratory showed that TNF-β signaling induces NF-κB activation, colony formation, epithelium-to-mesenchymal transition, CSCs formation and migration of CRC tumor cells in a tumor microenvironment [18,42,85]. We found that resveratrol inhibits the expression and activation of TNF-β and TNF-β-promoted inflammatory microenvironments on the survival and malignancy of colon cancer cells.

However, it should be noted that the low bioavailability and rapid metabolism of resveratrol in the body not only limits its therapeutic and biological effects, but also causes some changes in the way resveratrol is administered. In fact, numerous studies have been conducted to improve the bioavailability of resveratrol, and an important method of administering resveratrol is nano-based systems [87]. Interestingly, it has been reported that lipid nano-capsules loaded with resveratrol have higher anti-oxidant and anti-inflammatory effects than resveratrol alone [88]. In addition, the currently available known data on resveratrol are not sufficiently plausible to recommend the administration of resveratrol to humans beyond long-term doses. The precise administration of resveratrol, with its limitation to long-term doses, should be possible until longer clinical trials in humans have been conducted to assess the efficacy and safety of resveratrol.

**Figure 8:**
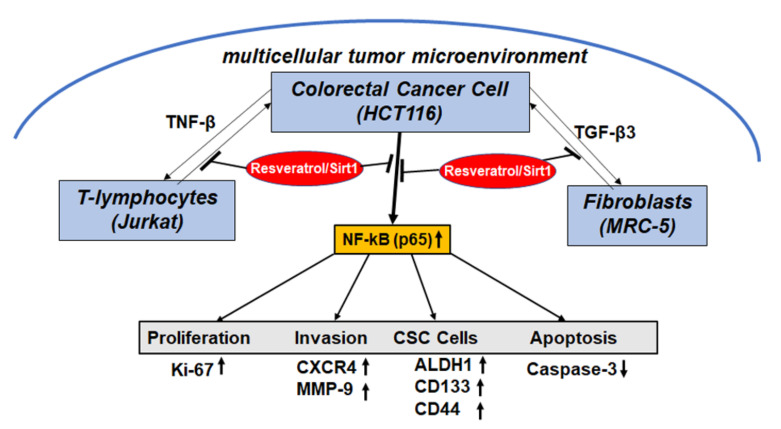
Multitargeting impacts of resveratrol on the malignancy of CRC cells in the multicellular-TME.

Overall, the current study showed for the first time that multiple molecular targets of resveratrol/Sirt1 signaling can suppress the growth and invasion of CRC tumors in pro-inflammatory multicellular-TME (Figure 8). This study on the preclinical, anti-metastatic effect of resveratrol in CRC gives an insight into the promising applications of resveratrol in the prevention, adjuvant and therapeutic treatment of colorectal cancer.

## 4. Materials and Methods

### 4.1. Antibodies and Chemicals

Antibodies to TGF-β3, p65-NF-κB, phospho-p65-NF-κB, MMP-9, CXCR4, and cleaved-caspase-3 were purchased from R&D Systems (Heidelberg, Germany), antibodies to TNF-β, ALDH1, CD44, CD133 were purchased from Antibodies online (Aachen, Germany), and antibodies to Sirt1 (sirtuin-1) were obtained from Cell Technology (Beverly, MA, USA). Ki-67 antibodies and rhodamine-coupled secondary antibodies for immunofluorescence were obtained from Dianova (Hamburg, Germany). Sheep anti-mouse and sheep anti-rabbit alkaline phosphatase linked secondary antibodies for Western blotting were from Millipore (Schwalbach, Germany). β-Actin antibodies, MTT reagent (3- (4,5-dimethylthiazol-2-yl)-2,5-diphenyltetrazolium bromide), DAPI, resveratrol (molecular weight 228.2, purity 98%) and alginate were obtained from Sigma-Aldrich (Taufkirchen, Germany). Resveratrol was prepared as a 100 mM stock in ethanol and further diluted in cell culture medium for experimental investigations. The final concentration of ethanol did not exceed 0.1% during the experiments.

### 4.2. Cell Lines and Cell Culture Conditions

A human colorectal cancer cell line, HCT116, and a normal human fibroblast cell line (MRC-5) were obtained from the European Collection of Cell Cultures (Salisbury, UK) and cultured as monolayers, and a human T-lymphocytes cell line (Jurkat) was purchased from the Leibniz Institute (DSMZ-German Collection of Microorganisms and Cell Cultures) and cells cultured in suspension. All cell lines were cultured under standard culture condition (37 °C, 5% CO_2_) with whole cell culture medium containing 10% FCS as previously described [89].

### 4.3. Experimental Setup

Colorectal cancer cells were cultured in combination with fibroblast cells and T-lymphocytes inflammatory cells in vitro to establish a multicellular tumor microenvironment (TME) to investigate the effect of resveratrol on modulating cross-talk between HCT116 cells and their TME (Figure 1).

To create the pro-inflammatory multicellular-TME, stromal fibroblast MRC-5 were cultured as a monolayer in petri dishes (3000/cm^2^) and HCT116 were encapsulated in 3-dimensional alginate bead cultures as outlined below and described in detail [86]. To establish the “multicellular-TME”, HCT116-alginate beads and Jurkat cells (20.000 cells/mL) were transferred to the petri dishes containing the MRC-5 monolayers and co-cultured in serum reduced cell culture medium (3% FCS). As a “basal control”, HCT116-alginate beads were cultured alone. Additionally, to compare the role of pro-inflammatory cytokine TNF-β in TME to natural pro-inflammatory multicellular-TME, a control culture without T-lymphocytes but instead with TNF-β was performed. These cultures were termed “TNF-β-TME” cultures. For the experiments, “basal control” and/or “TNF-β-TME” cultures were left untreated and “multicellular-TME” cultures were either left untreated or treated with various concentrations of resveratrol (1, 2, 5, 10 µM), or resveratrol 5 µM and either ASO-Sirt1 or SO-Sirt1 for 14 days. The culture medium with resveratrol and/or SIRT1-ASO or SIRT1-SO was changed every 3 days. All experiments were performed in serum-reduced cell culture medium containing 3% FCS.

### 4.4. Lipofectin Mediated Antisense Transfection

Transient knock-down of SIRT1 was performed with phosphonothioate antisense oligonucleotide derived from the mRNA nucleotide sequence of sirtuin-1 gene (Sirt1-ASO; sequence 5′-GTATTCCACATGAAACAGACA-3′) as described [36]. Antisense and control sense oligonucleotides (Sirt1-SO; sequence 5′-TGTCTGTTTCATGTGGAATAC-3′) were from Eurofins (MWG/Operon, Ebersberg, Germany). HCT116 in alginate beads were transfected with 0.5 µM Sirt1-ASO or Sirt1-SO and 10 µL/mL Lipofectin transfection reagent (Invitrogen) in serum-reduced cell culture medium (3% FCS) for the duration of the experiments. The results are provided as mean values from at least three independent experiments.

### 4.5. Alginate Culture, Colonosphere Formation and Invasion Assay

CRC cell line HCT116 cells were encapsulated in alginate 3-dimensional culture as described in [89,90]. In short, HCT116 cells (1 × 10^6^/mL) in sterile alginate solution (2% in 0.15 M NaCl) were added dropwise into a CaCl_2_ solution (100 mM) were they polymerized into beads. Before starting the experiments, beads were washed once with NaCl solution (0.15 M), twice with cell culture medium and were incubated for 1 h in serum reduced medium (3% FCS). Formation of colonospheres and invasion were assessed by light microscopy (Zeiss, Oberkochen, Germany) as shown previously [16]. Assessment of the number of colonospheres was performed by quantifying 20 microscopic fields. Invasive cells were assessed by staining (toluidine blue) and counting adhered colonies at the bottom of the petri dishes. All images were digitally stored.

### 4.6. Cell Proliferation Assay

Cell proliferation and vitality was assessed by the MTT method as described [18]. In short, after dissolving of the alginate beads in sterile sodium citrate solution (55 mM), cells were re-suspended in modified cell culture medium (without phenol red, without vitamin C, 3% FCS), and 100 µL with 10 μL MTT solution (5 mg/mL) per well distributed to a 96-well plate. After 3 h of incubation the reaction was stopped (10% Triton x-100/acidic isopropanol) and samples incubated overnight (37 °C). Finally, metabolically active cells were determined by measuring optical density with a revelation 96-well multiscanner plate ELISA reader (Bio-Rad Laboratories Inc. Munich, Germany).

### 4.7. Immunofluorescence

Sub-cellular localisation of NF-κB as a result to the above described treatment in multicellular-TME was investigated by immunofluorescence as described [86]. For immunofluorescence the multicellular-TME cultures were slightly modified: HCT116 were cultured in monolayer on glass plates (5000/glass plate), MRC-5 in monolayer on the bottom of the petri dish and T-lymphocytes in suspension. To prevent HCT116 on glass plates from directly lying on the MRC-5 monolayer, glass plates were placed on a steel net bridge in the petri dishes. Before the treatments were started, the pro-inflammatory multicellular-TME was allowed to develop for 24 h.

Glass plates were fixated for 15 min with methanol, washed twice with PBS/BSA 1% and incubated with primary antibodies (1:80) overnight in a humid chamber (4 °C). Samples were subsequently incubated with secondary rhodamine-coupled antibodies (1:100) for 2 h, and 15 min with DAPI to stain cell nuclei and embedded in Fluoromount (Sigma-Alderich, Munich, Germany). Images were acquired with a Leica DM2000 microscope (Wetzlar, Germany). A total of 500–600 cells in 25 microscopic fields were counted to quantify NF-κB staining as well as apoptosis.

### 4.8. Immunoblotting Assay

Western blot investigations were performed with whole cell lysates as described in [89]. After dissolving of the alginate, HCT116 cells were lysed (50 mM Tris/HCl, pH 7.2/150 mM NaCl/(*v*/*v*) Triton x-100/1 mM sodium orthovanadate/50 mM sodium pyrophosphate/100 mM sodium fluoride/4 µg/mL pepstatin A/1 mM PMSF) on ice for 30 min and total protein content was measured with the bicinchinonic acid system (Uptima, Monlucon, France) using bovine serum albumin as standard. After reduction, proteins were separated by SDS-PAGE electrophoresis, and blotted onto a nitrocellulose membrane (Transblot apparatus, Bio-Rad, Munich, Germany). Membranes were incubated for 2 h with primary antibodies (1:10.000) in blocking buffer (skimmed milk powder 1% in PBS) and for 1.5 h with secondary antibodies (1:10.000) and the specific binding detected using nitro blue tetrazolium and 5-bromo-4-chloro-3-indoyl-phosphate (VWR, Munich, Germany). The bands were quantified by the Quantity One program (Bio-Rad, Munich, Germany) and β-Actin was used to normalize samples to control.

### 4.9. Statistical Evaluation

For statistical evaluation, all experiments were performed at least three times as single assays and a Wilcoxon–Mann–Whitney test was applied. The results were presented as mean + SD or SEM and compared by one-way, two-way or three-way ANOVA using SPSS Statistics if the normality test was passed (Kolmogorov–Smirnov test) and a *p*-value of <0.05 was regarded as showing statistically significant differences.

## 5. Conclusions

Our results suggest that multicellular-TME enhances CRC malignancy, and this is at least partially mediated by the secretion of TNF-β and TGF-β3, which in turn leads to activation of NF-κB signaling, inducing new cross-talk between stomal cells and HCT116 cells in the CRC TME. Interestingly, the down-modulation of active cross-talk in the multicellular-TME by resveratrol interrupts this loop and by modulating the resveratrol-Sirt1 axis signal, significantly reduces HCT116 cell survival, migration and CSC-mediated metastasis (Figure 8).
